# Favorable Climate Change Response Explains Non-Native Species' Success in Thoreau's Woods

**DOI:** 10.1371/journal.pone.0008878

**Published:** 2010-01-26

**Authors:** Charles G. Willis, Brad R. Ruhfel, Richard B. Primack, Abraham J. Miller-Rushing, Jonathan B. Losos, Charles C. Davis

**Affiliations:** 1 Department of Organismic and Evolutionary Biology, Harvard University Herbaria, Cambridge, Massachusetts, United States of America; 2 Department of Biology, Duke University, Durham, North Carolina, United States of America; 3 Department of Biology, Boston University, Boston, Massachusetts, United States of America; 4 USA National Phenology Network, Tucson, Arizona, United States of America; 5 The Wildlife Society, Bethesda, Maryland, United States of America; 6 Department of Organismic and Evolutionary Biology, Museum of Comparative Zoology, Cambridge, Massachusetts, United States of America; Centre National de la Recherche Scientifique, France

## Abstract

Invasive species have tremendous detrimental ecological and economic impacts. Climate change may exacerbate species invasions across communities if non-native species are better able to respond to climate changes than native species. Recent evidence indicates that species that respond to climate change by adjusting their phenology (i.e., the timing of seasonal activities, such as flowering) have historically increased in abundance. The extent to which non-native species success is similarly linked to a favorable climate change response, however, remains untested. We analyzed a dataset initiated by the conservationist Henry David Thoreau that documents the long-term phenological response of native and non-native plant species over the last 150 years from Concord, Massachusetts (USA). Our results demonstrate that non-native species, and invasive species in particular, have been far better able to respond to recent climate change by adjusting their flowering time. This demonstrates that climate change has likely played, and may continue to play, an important role in facilitating non-native species naturalization and invasion at the community level.

## Introduction

Invasive species have significant negative effects on biodiversity, ecosystem function, agricultural productivity, and human health [1]. In the United States alone the estimated annual cost of invasive species exceeds $120 billion [2]. It has been hypothesized that climate change will exacerbate the impacts of non-native species naturalization and subsequent invasion across communities [3], [4], [5], [6]. One important way in which non-native species could respond to climate change is by adjusting their phenology (i.e., the timing of seasonal activities, such as flowering time, leaf out time, germination and migration) [7], [8]. Along these lines, the ability of species to appropriately adjust their phenology to climate change has been shown to have a significant impact on species success [9], [10], [11]. The extent to which phenological response is also linked to non-native species success, however, has not been examined, despite its potential relevance to conservation and management efforts in the face of continued climate change.

Here, we take advantage of a unique historical dataset from Concord, Massachusetts (USA) [12] to elucidate the role of climate change in shaping the patterns of non-native plant species' naturalization and invasion (see Material and Methods). Concord has experienced significant climate change in the last 150 years, during which time the mean annual temperature has increased 2.4°C [12]. The Concord dataset was initiated in 1851 by the American naturalist and conservationist Henry David Thoreau and continued by later observers, including the authors of this paper [12]. This dataset permits the calculation of two important phenological response traits: i) the ability of species to track seasonal temperature variation measured as the correlation between first flowering day and annual spring temperature from 1888–1902 (herein referred to as *flowering time tracking*) and ii) the change in mean first flowering day over two periods: 1851–2006 and 1900–2006 (herein referred to as *flowering time shift*, see Material and Methods).

We distinguished between native and non-native species using the United States Department of Agriculture (USDA) PLANTS Database [13]. We further distinguished non-native non-invasive and non-native invasive (i.e., herein referred to as invasive) species using the Invasive Plant Atlas of New England [14] (for complete definitions of non-native species status see Material and Methods). To account for other factors that could also explain non-native species success, we examined several additional ecologically important traits that have been implicated in non-native species' naturalization and invasion [15], including: habit, plant height at maturity, leaf mass per area, flower diameter, pollination syndrome, and seed weight.

We tested for significant differences in these traits between: i.) natives and non-natives, ii.) natives and non-native non-invasives, iii.) natives and invasives, and iv.) non-native non-invasives and invasives. These comparisons were tested using generalized estimating equations (GEE) implemented in the R based package APE [16], [17]. GEE allow phylogenetic distance matrices to be incorporated into a general linear model framework so as to account for phylogenetic bias and permits the simultaneous analysis of multiple categorical and continuous traits as covariates in the same model [17].

## Results and Discussion

**Figure 1:**
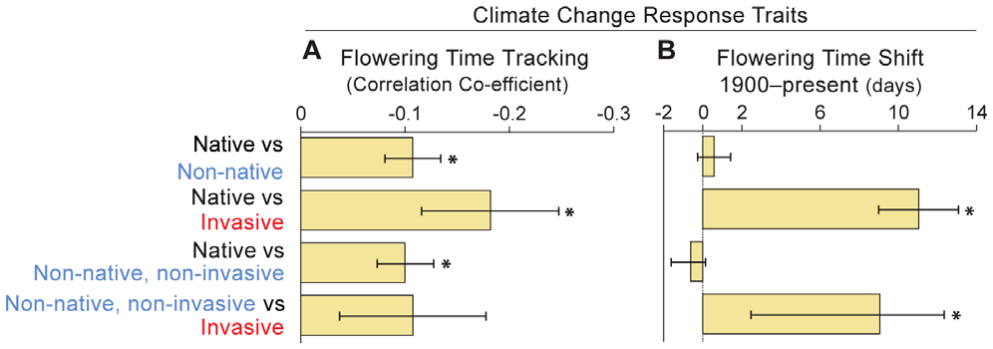
Bar graphs depicting phylogenetically corrected mean differences between species groups for two climate change response traits: the correlation coefficient between first flowering day and annual spring temperature for the time period of 1888–1902 (A; i.e., *flowering time tracking*), and the shift in mean first flowering day during the period exhibiting the most dramatic increase in mean annual temperature, from 1900–2006 (B; i.e., *flowering time shift*). Trait differences significantly greater than zero are indicated with an asterisk (p≤0.05). Error bars indicate standard errors.

Our results indicate that non-native species differ dramatically from natives in their ability to respond to climate change (Fig. 1, Table 1, Table S1). Non-natives are significantly better able to track seasonal temperatures than native species (Fig. 1A, Table 1). In particular, invasives track seasonal temperature variation better than natives and non-native non-invasives, although the difference between invasives and non-native non-invasives is not significant. Invasives have also significantly shifted their flowering time over the last 100 years to be 11 days earlier than natives and 9 days earlier than non-native non-invasives (Fig. 1B; results are also similar for the 1851–2006 time interval, see Table 1). Concordant with these phenological results, non-native and particularly invasive species have significantly increased in abundance since 1900 relative to the native flora (Table 1). Finally, aside from having slightly larger flowers than natives, which is likely due to the fact that many non-natives are escaped ornamentals [18], non-native species showed no appreciable difference in the other traits we examined (Table 1).

**Table 1:** Trait correlations with non-native status.

				Non-native vs. Native	Invasive vs. Native	Non-native non-invasive vs. Native	Invasive vs. Non-native non-invasive
traits	n_1_	n_2_	n_3_	β-coefficient	β-coefficient	β-coefficient	β-coefficient
Change in abundance (1900–2006)	260	69	15	1.26±0.10***	2.39±0.20***	1.03±0.73***	1.56±0.30***
Flower diameter	372	129	34	0.07±0.02**	0.02±0.04	0.08±0.02***	−0.06±0.04
Flowering time shift (1851–2006)	245	52	8	−3.11±1.01**	9.98±2.64***	−4.12±1.00***	10.89±3.74**
Flowering time shift (1900–2006)	245	65	11	0.60±0.84	11.04±2.04***	−0.70±0.87	9.07±3.25*
Flowering time tracking	126	25	5	−0.11±0.03***	−0.18±0.07**	−0.10±0.03***	−0.11±0.07
Habit (herb v. woody)	256	97	23	0.004±0.01	0.01±0.02	0.003±0.01	0.01±0.01
Height at maturity	336	80	16	−0.02±0.03	0.08±0.07	−0.03±0.03	0.09±0.06
Leaf mass per area	53	39	11	0.01±0.03	0.01±0.03	0.07±0.05	0.02±0.04
Seed weight	275	123	31	0.10±0.05†	0.03±0.10	0.11±0.06*	−0.07±0.09
Syndrome (insect v wind)	385	136	35	−0.01±0.01	0.002±0.02	−0.01±0.01	0.002±0.02
Non-native Status	385	136	35	–	–	–	–
Invasive Status	385	136	35	–	–	–	–

Trait correlations between groups were tested using general estimator equations (GEE). Results shown here are robust to branch length estimates and phylogenetic uncertainty (see also Table S1). β-coefficients describe the direction and magnitude of the difference between groups. For example, a β-coefficient of -0.11 for flowering time tracking indicates that non-natives have a significantly greater negative correlation between flowering time and seasonal temperature variation than natives. Standard error of β-coefficients provided. n = sample size of 1) natives, 2) non-native non-invasives and 3) invasives. † P<0.07; * P<0.05; ** P<0.01, *** P<0.001.

These results provide the strongest link to date between climate change and non-native species' naturalization and subsequent invasion at the community level. While the evolutionary and ecological mechanisms for these results require further investigation, our results nevertheless highlight the utility of phenological response as an important tool for assessing the likelihood of future naturalizations and subsequent invasions by non-native species. Specifically, these results indicate that information on flowering time tracking may allow us to determine if a non-native species is more likely to become naturalized in its introduced range. In addition, the likelihood that a non-native species will become invasive will benefit most from data on species flowering time shift. For example, in Concord, mayweed chamomile (*Anthemis cotula* L.) has greatly shifted its flowering time 23 days earlier since 1900. While mayweed chamomile has yet to be classified as an invasive in Massachusetts, our results from above suggest that it has a high potential of becoming invasive with continued climate change.

In summary, our study indicates that non-native species possess a common set of phenological traits that have likely facilitated their success in the face of recent climate change. As climate change accelerates, non-native species' ability to respond favorably will likely exacerbate the ecological and economic problems that result from their success. Moreover, because climate change affects large geographical regions in a similar manner, its impact on non-native species naturalization and invasion could be more pervasive than other global change factors that act more regionally (e.g., increasing nitrification, habitat disturbance, and underground microbial species composition) [5], [6]. To what extent non-native species have exhibited similar climate change responses in other communities, however, is limited by the rarity of long-term community datasets that document species' phenological responses [19]. Future efforts should be focused on expanding the documentation of species' phenological response data through direct observation of phenology [19], [20], historical records [21], observations of pollinators [22], experimental manipulation [23], quantitative genetic techniques [24] and comparative studies [10], [25], [26]. These data will likely be essential for assessing and managing the future impacts of invasive species in the face of continued climate change.

## Materials and Methods

### Study Site

Concord, Massachusetts, USA (42°27′38″ N; 71°20′54″ W) is a township encompassing ∼67 km^2^. Although the town has undergone extensive development since the time of Henry David Thoreau (∼1850), ∼60% of Concord remains undeveloped or has been well protected through the efforts of numerous national, state, local, private parks, and land-trusts [27].

### Species' Native/Non-Native Status

Species' native and non-native status was obtained from the USDA PLANTS Database [13] for the 587 species included in our analyses. Species were scored as ‘native’ if they occurred in the continental United States or Canada at the time of Columbus (ca. 1492) and ‘non-native’ if they arrived from other regions since that time. Thirty-one species that were coded ambiguously by the USDA as ‘native and probably introduced’ were not included. When these species were coded as either ‘non-native’ or ‘native’ the results were not qualitatively different than those presented here. All non-native species were considered ‘naturalized’ because they are thought to be established members of the Concord flora [27].

Non-native species were further categorized as ‘non-native non-invasive’ and ‘invasive’ using the Invasive Plant Atlas of New England (IPANE) [14]. IPANE defines a non-native species as ‘invasive’ if it meets all of the following criteria: 1) the species is or has the potential to become naturalized in New England, 2) the species is or has the potential to establish in minimally managed habitats, 3) the species does or has the potential to disperse rapidly and widely, 4) the species does or has the potential to establish large populations in minimally managed habitats, and 5) the species is classified as invasive in other areas outside of its native range. Importantly, our analyses, which include data on change in abundance [10], independently corroborate IPANE's scoring of invasive species status. Invasive species, as classified by IPANE, have significantly increased in abundance in Concord relative to native and non-native non-invasive species over the past 100 years (Table S1).

### Ecological Trait Data

Ecological trait data was collected from multiple sources. Shift in mean first flowering day (1851–2006, 1900–2006), the correlation between flowering time and inter-annual temperature variation from 1888–1902 (i.e., *flowering time tracking*), and change in abundance were all calculated directly from observations of the Concord community [10], [12]. Flowering time tracking was calculated as the correlation between first flowering day and mean monthly temperature in January, April, and May. This aggregate temperature was determined to be the best predictor flowering day in Concord [28], [29]. We also obtained several additional ecologically relevant traits that have been implicated in non-native species success, including: leaf mass per area [30], plant height at maturity [13], seed weight [31], habit [32], flower diameter [32], [33], and pollination syndrome [32], [33]. Habit was coded as a binary trait (herbaceous vs. woody) using the *Manual of the Vascular Plants of Northeastern United States and Adjacent Canada* [32]. Pollination syndrome was coded as a binary trait (i.e., wind vs. insect pollinated) using refs [32], [33], [34]. Binary traits were treated as continuous in our correlation analyses and results should be interpreted as relative proportions. Leaf mass per area, plant height, seed weight, and flower diameter were log-transformed when necessary to meet the assumptions of normality.

This study focuses on the ability of species to adjust their flowering phenology in response to climate change, an ability that has been shown to have important implications for species success [10]. Although poorly understood, a species' ability to succeed might be linked with flowering time shifting if its fitness was directly dependent on when it flowered. For example, species that are unable to appropriately adjust their flowering time in response to climate change could suffer from a sudden lack of pollinators [22], [35]. Alternatively, species' fitness could be indirectly dependent on flowering time. For instance, changes in flowering time are generally correlated with the timing of leaf out, a character that is often linked to competitive ability and physiological adaptation [36], [37], [38]. A species that starts its growing cycle earlier in warmer years could have a competitive advantage in terms of nutrient acquisition and light availability. As a result, they may be more likely to complete their life cycle under favorable conditions (e.g., before the onset of warm and dry weather during the middle to late summer). Furthermore, a species that is able to leaf out earlier in warmer years could potentially shade out co-occurring species that are not responsive to temperature. Similarly, a species that puts out its leaves later in cold years might avoid late frosts that could damage its leaves.

### Trait Correlations

Standard trait correlations may be biased by species relatedness [39]. To account for evolutionary history in assessing trait correlations, we used the generalized estimating equations as implemented in APE [16], [17]. GEE incorporates a phylogenetic distance matrix into the framework of a general linear model and permits the inclusion of multiple categorical and continuous traits as covariates in the same model [17]. This is similar to normal general linear regression in that the β-coefficient describes the direction and magnitude of the difference between groups (e.g., the directional difference in shift response between native and non-native species). Our conclusions are drawn here from the results obtained from GEE analysis (Table S1), but correlation results were similar when using phylogenetic independent contrasts as implemented in the ‘aotf’ module in Phylocom [40].

### Phylogeny Construction

We constructed an initial composite phylogeny of the Concord flora using Phylomatic [41] and further resolved relationships above the generic level based on the literature. For complete details of our construction of the phylogeny see Willis *et al.* [10]. Divergence time estimates were calculated using the ‘bladj’ function in Phylocom [40] based on Wikström *et al.* [42].

### Sensitivity Analysis

Phylogenetic correlations can be biased by branch length estimates and phylogenetic resolution. We tested the sensitivity of our results to branch length estimates by setting all branch lengths equal to one. We tested the sensitivity of our results to phylogenetic uncertainty by performing our analyses on a set of 50 phylogenies where all polytomies, above and below the generic level, were randomly resolved using the ‘multi2di’ function in APE and ages were re-estimated using the ‘bladj’ function in Phylocom. Regression results were robust to both of these sensitivity analyses (Table S1).

### Multivariate Analyses Including Abundance Data

Flowering time shift results remained similar when change in abundance [43] was included as an independent variable in the models we analyzed (*flowering time shift 1851–2006*: Non-native vs Native, β = −3.61, SE = 1.02, t = −3.54, p = 0.0007; Invasive vs Native, β = 8.28, SE = 2.67, t = 3.10, p = 0.0029; Non-native non-invasive vs Native, β = −4.51, SE = 1.01, t = −4.48, p<.0001; Invasive vs Non-native non-invasive β = 11.30, SE = 3.81, t = 2.96, p = 0.0090; *flowering time shift 1900–2006*: Non-native vs Native, β = −0.10, SE = 0.84, t = −0.12, p = 0.9015; Invasive vs Native, β = 9.50, SE = 2.05, t = 4.64, p<.0001; Non-native, non-invasive vs Native, β = -1.26, SE = 0.87, t = −1.46, p = 0.1498; Invasive vs Non-native, non-invasive, β = 7.99, SE = 3.23, t = 2.47, p = 0.0216).

## Supporting Information

Table S1 Statistical tests of trait correlations.
(0.34 MB DOC)
