# Supplementary material for: Favorable Climate Change Response Explains Non-Native Species' Success in Thoreau's Woods
Source: PLoS One. 2010 Jan 26;5(1):e8878. doi: 10.1371/journal.pone.0008878 (PMC2811191; doi:10.1371/journal.pone.0008878)
Supplement: Supplementary file 1 [file pone.0008878.s001.doc]

					Non-native vs. Native				Invasive vs. Native				Non-native non-invasive vs. Native				Invasive vs. Non-native non-invasive			
	traits	n_1	n_2	n_3	β-coeff	S.E.	t	p-value	β-coeff	S.E.	t	p-value	β-coeff	S.E.	t	p-value	β-coeff	S.E.	t	p-value
Table 1A	Charge in abundance 1900-2006	260	69	15	1.26	0.10	12.42	<.0001	2.39	0.20	11.87	<.0001	1.03	0.73	9.56	<.0001	1.56	0.30	5.16	<.0001
	Flower diameter	372	129	34	0.07	0.02	3.40	0.0010	0.02	0.04	0.43	0.6682	0.08	0.02	3.74	0.0003	-0.06	0.04	-1.57	0.1248
	Flowering time shift 1851-2006	245	52	8	-3.11	1.01	-3.07	0.0031	9.98	2.64	3.78	0.0003	-4.12	1.00	-4.13	0.0001	10.89	3.74	2.91	0.0096
	Flowering time shift 1900-2006	245	65	11	0.60	0.84	0.72	0.4759	11.04	2.04	5.41	<.0001	-0.70	0.87	-0.81	0.4212	9.07	3.25	2.79	0.0103
	Flowering time tracking	126	25	5	-0.11	0.03	-4.12	0.0002	-0.18	0.07	-2.78	0.0079	-0.10	0.03	-3.73	0.0003	-0.11	0.07	-1.53	0.1484
	Habit (herb v. woody)	256	97	23	0.00	0.01	0.37	0.7143	0.01	0.02	0.35	0.7287	0.00	0.01	0.30	0.7664	0.01	0.01	0.50	0.6237
	Height at maturity	336	80	16	-0.02	0.03	-0.46	0.6495	0.08	0.07	1.13	0.2631	-0.03	0.03	-0.85	0.3969	0.09	0.06	1.42	0.1678
	Leaf mass per area	53	39	11	0.01	0.03	0.50	0.6191	0.01	0.03	0.21	0.8363	0.07	0.05	1.36	0.1829	0.02	0.04	0.56	0.5811
	Seed weight	275	123	31	0.10	0.05	1.86	0.0659	0.03	0.10	0.28	0.7772	0.11	0.06	2.02	0.0466	-0.07	0.09	-0.77	0.4466
	Syndrome (insect v wind)	385	136	35	-0.01	0.01	-1.11	0.2686	0.00	0.02	-0.14	0.8928	-0.01	0.01	-1.22	0.2234	0.00	0.02	0.12	0.9087
Table 1B	Charge in abundance 1900-2006	260	69	15	1.40	0.10	14.29	<.0001	2.10	0.19	11.20	<.0001	1.23	0.11	11.53	<.0001	0.92	0.30	3.07	0.0064
	Flower diameter	372	129	34	0.07	0.02	4.15	0.0001	0.01	0.03	0.18	0.8616	0.09	0.02	4.87	<.0001	-0.10	0.04	-2.38	0.0158
	Flowering time shift 1851-2006	245	52	8	-0.61	1.18	-0.51	0.6097	10.14	2.86	3.54	0.0010	-1.71	1.17	-1.46	0.1313	9.42	3.63	2.59	0.0214
	Flowering time shift 1900-2006	245	65	11	1.42	0.87	1.64	0.1087	9.57	1.85	5.17	<.0001	0.00	0.89	0.00	0.9965	5.71	3.01	1.90	0.0745
	Flowering time tracking	126	25	5	-0.12	0.03	-3.68	0.0010	-0.08	0.08	-1.02	0.3186	-0.12	0.03	-3.69	0.0010	0.05	0.07	0.74	0.4753
	Habit (herb v. woody)	256	97	23	0.00	0.01	0.22	0.8285	0.01	0.01	0.42	0.6757	0.00	0.01	0.09	0.9263	0.01	0.01	0.51	0.6135
	Height at maturity	336	80	16	0.02	0.03	0.73	0.4699	0.18	0.06	2.98	0.0044	-0.01	0.03	-0.40	0.6900	0.14	0.06	2.34	0.0307
	Leaf mass per area	53	39	11	0.10	0.03	3.05	0.0055	0.12	0.05	2.43	0.0235	0.09	0.03	2.80	0.0102	-0.01	0.03	-0.19	0.8557
	Seed weight	275	123	31	0.16	0.05	3.25	0.0021	0.15	0.08	1.71	0.0983	0.16	0.05	3.13	0.0029	-0.02	0.08	-0.27	0.7877
	Syndrome (insect v wind)	385	136	35	0.00	0.01	0.32	0.7502	0.01	0.01	0.42	0.6757	0.00	0.01	0.09	0.9263	0.01	0.02	0.77	0.4473
Table 1C	Charge in abundance 1900-2006	260	69	15	1.19	0.11	11.39	<.0001	2.33	0.21	11.27	<.0001	0.96	0.11	8.69	<.0001	1.44	0.30	4.83	0.0002
	Flower diameter	372	129	34	0.06	0.02	3.39	0.0035	0.02	0.03	0.46	0.6234	0.07	0.02	3.72	0.0018	-0.07	0.04	-1.69	0.1166
	Flowering time shift 1851-2006	245	52	8	-2.30	1.03	-2.24	0.1465	10.64	2.56	4.19	0.0012	-3.45	1.02	-3.37	0.0534	10.93	3.66	3.00	0.0218
	Flowering time shift 1900-2006	245	65	11	0.50	0.89	0.56	0.4167	13.55	2.17	6.26	<.0001	-1.08	0.92	-1.18	0.3438	13.46	3.46	3.91	0.0035
	Flowering time tracking	126	25	5	-0.11	0.03	-4.22	0.0003	-0.19	0.07	-2.94	0.0138	-0.10	0.03	-3.82	0.0010	-0.12	0.07	-1.71	0.1766
	Habit (herb v. woody)	256	97	23	0.01	0.01	0.83	0.4035	0.01	0.02	0.43	0.6541	0.01	0.01	0.81	0.4233	0.01	0.01	0.47	0.6452
	Height at maturity	336	80	16	-0.01	0.03	-0.29	0.5376	0.09	0.07	1.28	0.2578	-0.03	0.04	-0.77	0.4377	0.06	0.06	1.04	0.3483
	Leaf mass per area	53	39	11	0.08	0.03	3.06	0.0054	0.11	0.04	2.59	0.0164	0.07	0.03	2.76	0.0111	-0.01	0.03	-0.19	0.8557
	Seed weight	275	123	31	0.09	0.05	1.84	0.0915	0.06	0.09	0.65	0.5347	0.10	0.05	1.88	0.0871	-0.06	0.09	-0.70	0.5256
	Syndrome (insect v wind)	385	136	35	-0.01	0.01	-1.19	0.3088	0.00	0.02	-0.08	0.8282	-0.01	0.01	-1.34	0.2617	0.00	0.02	0.21	0.7799
Table 1D	Charge in abundance 1900-2006	260	69	15	1.42	0.11	12.69	<.0001	2.51	0.21	12.03	<.0001	1.19	0.12	10.04	<.0001	RD	RD	RD	RD
	Flower diameter	372	129	34	0.08	0.02	4.47	<.0001	0.10	0.02	4.80	<.0001	0.10	0.02	4.80	<.0001	RD	RD	RD	RD
	Flowering time shift 1851-2006	245	52	8	-1.61	1.07	-1.51	0.1367	10.77	2.59	4.15	0.0001	-2.61	1.06	-2.45	0.0170	RD	RD	RD	RD
	Flowering time shift 1900-2006	245	65	11	4.39	0.94	4.67	<.0001	12.68	2.05	6.18	<.0001	3.07	0.99	3.10	0.0029	RD	RD	RD	RD
	Flowering time tracking	126	25	5	-0.07	0.03	-2.32	0.0253	-0.14	0.07	-2.05	0.0465	-0.06	0.03	-2.04	0.0483	RD	RD	RD	RD
	Habit (herb v. woody)	256	97	23	0.01	0.01	0.64	0.5228	0.01	0.02	0.42	0.6741	0.01	0.01	0.60	0.5501	RD	RD	RD	RD
	Height at maturity	336	80	16	-0.04	0.04	-1.25	0.2134	0.05	0.08	0.71	0.4811	-0.06	0.04	-1.61	0.1123	RD	RD	RD	RD
	Leaf mass per area	53	39	11	RD	RD	RD	RD	RD	RD	RD	RD	RD	RD	RD	RD	RD	RD	RD	RD
	Seed weight	275	123	31	0.07	0.06	1.24	0.2191	0.01	0.10	0.11	0.9109	0.08	0.06	1.38	0.1699	RD	RD	RD	RD
